# Understanding the acceptability of COVID-19 antigen rapid diagnostic tests: A multi-country qualitative study

**DOI:** 10.1371/journal.pgph.0005119

**Published:** 2025-11-24

**Authors:** Wezzie S. Lora, Yasmin Dunkley, Itai Kabonga, Elvis Isere, MackWellings Phiri, Chimwemwe Kwanjo Banda, Euphemia Sibanda, John Bimba, Richard Chilongosi, Karin Hatzold, Frances M. Cowan, Elizabeth L. Corbett, Augustine T. Choko, Nicola Desmond

**Affiliations:** 1 Malawi Liverpool Wellcome Trust Clinical Research Programme, Blantyre, Malawi; 2 Liverpool School of Tropical Medicine, Liverpool, United Kingdom; 3 London School of Hygiene and Tropical Medicine, London, United Kingdom; 4 Centre for Sexual Health and HIV AIDS Research, Harare, Zimbabwe; 5 Zankli Research Centre, Bingham University, Abuja, Nigeria; 6 Kamuzu College of Health Sciences, Blantyre, Malawi; 7 Family Health Services, Blantyre, Malawi; 8 Population Services International, Cape Town, South Africa; University of Oxford, UNITED KINGDOM OF GREAT BRITAIN AND NORTHERN IRELAND

## Abstract

Accessible and timely diagnostic testing was essential during the COVID-19 pandemic to control the virus’s spread and mitigate severe outcomes. However, real-time polymerase chain reaction tests, although sensitive incur high costs and prolonged turnaround times, and face implementation challenges in many resource-constrained settings. Antigen rapid diagnostic tests (Ag-RDT), which can be administered either by providers or as self-tests, are accessible and low-cost alternatives. We conducted this study to assess the acceptability of Ag-RDT in different use cases and delivery models in Malawi, Nigeria, and Zimbabwe. We implemented a qualitative study drawing on Louart’s theoretical framework for the acceptability of technological healthcare innovations. In-depth interviews (n = 228) were conducted with community members, healthcare workers and providers to explore participants’ experiences with Ag-RDT COVID-19 testing. Data were analysed using NVivo 14 software, applying an inductive thematic approach centred on the constant comparison method across countries, participant groups, and delivery models. The study revealed that Ag-RDT COVID-19 testing was acceptable across the three settings due to speed, non-invasiveness, and simplicity in sample collection compared to the real-time polymerase chain reaction alternative. Ag-RDT was integrated into existing healthcare systems with minimal disruption and used effectively across different use cases and populations. Participants particularly valued the self-testing option for its privacy, reduced stigma and convenience. However, acceptability was somewhat affected by the timing of the intervention, as it was implemented toward the end of the COVID-19 pandemic when many individuals no longer perceived testing as a priority. COVID-19 Ag-RDT testing was an acceptable complementary methodology to PCR testing in Malawi, Nigeria and Zimbabwe. The findings emphasise the need for strategic investment in accessible diagnostic technologies that complement existing molecular diagnostics, particularly supporting decentralised testing and rapid response capabilities in resource-constrained settings while maintaining robust surveillance systems.

## Introduction

During the COVID-19 pandemic, accessible and timely diagnostic testing was essential in controlling the virus’s spread and mitigating severe health outcomes. Real-time polymerase chain reaction (RT-PCR) and other nucleic acid amplification tests (NAAT), while highly sensitive, require substantial infrastructure and trained personnel [1–3]. In low-and middle-income countries (LMIC), particularly in sub-Saharan Africa (SSA), limited testing capacity, high user costs, lengthy distances to laboratories, and prolonged result turnaround times present significant barriers to effective testing and control efforts.

Antigen rapid diagnostic tests (Ag-RDT) are accessible, low-cost alternatives, particularly suited for LMIC contexts where immediate diagnosis can aid optimised clinical management, prevention efforts, and support identification of new outbreaks, including potential variants [4,5]. Their simplicity and low cost make Ag-RDT suitable for serial testing strategies and contact tracing, offering a flexible and potentially scalable approach to managing outbreaks [6]. Ag-RDT can be provider-delivered or self-administered. Self-testing allows individuals to conduct tests at home, supporting rapid diagnosis and isolation where professional healthcare access is limited [7,8]. Evidence from HIV self-testing in Southern Africa has shown that factors like ease of use, perceived accuracy, privacy, confidentiality, and perceived risk of harm are critical to client acceptability, while healthcare providers’ acceptance depends on accuracy, cost-effectiveness, and resource availability [9–12].

Although the World Health Organization (WHO) no longer classifies COVID-19 as a pandemic, resurgence threats exist, and future infectious disease outbreaks are anticipated [13]. Ag-RDT will be crucial in managing potential COVID-19 resurgences and a valuable complimentary diagnostic tool for various emerging infections. This adaptation of testing technology demonstrates how lessons learned from COVID-19 can inform broader infectious disease surveillance and control strategies [14]. The situation is particularly critical in LMIC, where healthcare systems operate under severe resource constraints affecting the comprehensive pandemic preparedness and response capacity. Understanding Ag-RDT acceptability under dynamic epidemiological conditions and balancing self-testing and provider-delivered testing is crucial for shaping adaptable and effective Ag-RDT implementation strategies that address the unique and fluctuating demands of different populations and outbreak scenarios.

Studies in Southern Africa have demonstrated Ag-RDT feasibility but have not explored its acceptability in-depth [15,16]. Questions remain regarding COVID-19 Ag-RDT acceptability in LMIC, where diagnostic preferences and challenges vary widely by context and resource availability. This paper explores the acceptability of COVID-19 Ag-RDT, including self-testing and provider-delivered testing in Malawi, Nigeria and Zimbabwe. These countries represent diverse healthcare infrastructures, cultural contexts, and economic conditions across SSA [17–19]. Malawi and Zimbabwe had strong community health programs but limited laboratory infrastructure and poor rural access [20–22], while Nigeria’s larger, more diverse population utilised mobile technology for case tracking but faced persistent misinformation challenges [23]. The study targeted different populations and employed various use cases for implementing COVID-19 Ag-RDT, allowing for a comparative assessment of their acceptability in diverse environments. This diversity makes the study particularly relevant for informing Ag-RDT implementation strategies and tailoring interventions to suit various settings.

### Theoretical framework

**Fig 1:**
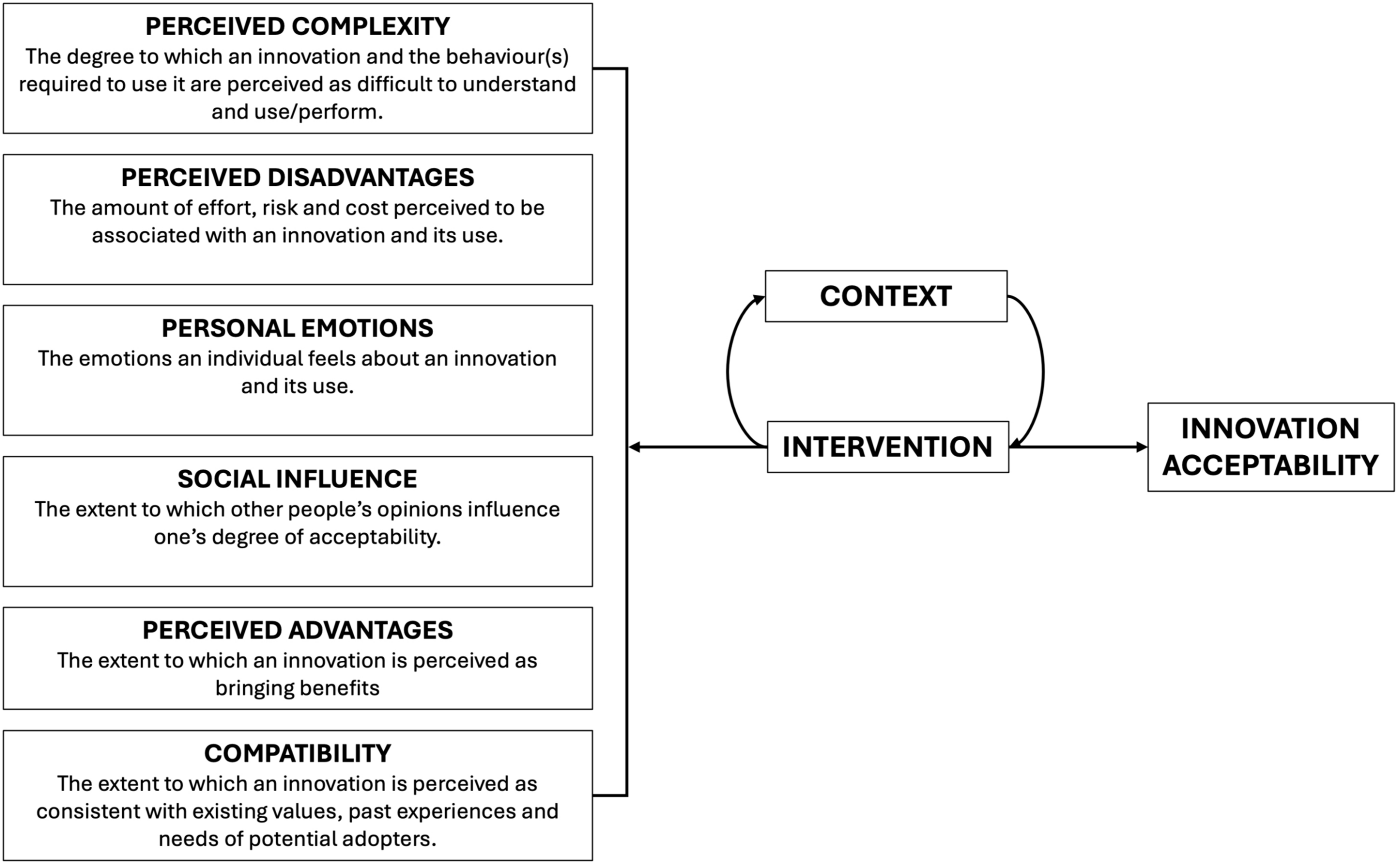
Framework for the acceptability of technological health innovations [25].

We drew on Lourt’s theoretical framework on the acceptability of technological healthcare innovations (See Fig 1 for the framework) [24]. This framework was selected because it is tailored to understand the acceptability of technological health innovations, such as diagnostic tools. Acceptability is defined as how an actor reacts to a technological innovation shaped by multiple factors that collectively influence the decision to use (and/or agree to use) the technological innovation in health [25]. Louart’s framework examines innovations from both user and system perspectives, focusing on six determinants of acceptability: compatibility, perceived complexity, perceived advantages, perceived disadvantages, personal emotions and social influence. We tested the extent to which Louart’s theory applied to our findings, demonstrating its relevance and breaking it down by each domain. The acceptability of Ag-RDT was evaluated during its implementation phase [26,27].

## Methods

We employed a cross-sectional qualitative design between April 2022 and June 2023. In-depth Interviews (IDIs) were used to collect data on the experiences, varying opinions, and future outlook regarding the Ag-RDT [28]. The research procedures and reporting followed the consolidated criteria for reporting qualitative research [29].

### Ethics approval and consent to participate

#### Malawi.

All study procedures were approved by Kamuzu University of Health Science’s College of Medicine Research and Ethics Committee (COMREC) in Malawi under the number P.05/22/3649, and internationally from the WHO Ethics Review Committee in Geneva, Switzerland under the number CERC.0163, and the London School of Hygiene & Tropical Medicine Ethics Committee under the number 26874.

#### Nigeria.

The study was approved by the the Federal Capital Territory Authority (FCTA), Abuja, Ethical Review Committee, Nigeria with protocol number: FHREC/2022/01/29/09-03-22, the London School of Tropical Medicine and Hygiene Intervention Research Ethics Committee (protocol ID: 26886) and the WHO Ethical Review Committee (protocol ID: CERC.0165).

#### Zimbabwe.

The study was approved by Medical Research Council of Zimbabwe (MRCZ), under the number MRCZ A2872, London School of Hygiene and Tropical Medicine (LSTM), under the number 26931 and the WHO Ethics Review Committee (ERC), under the number CERC.0160.

All participants provided written informed consent at enrolment. All quotes have been labelled with identification numbers to protect participants’ identities. Consent for publication is not applicable.

### Study context

This study was nested within the Africa, Asia, Americas COVID-19 Preparedness (3ACP) Consortium which aimed to test and optimise different use cases (participant groups representing healthcare and community contexts regardless of their COVID-19 testing outcomes) for delivering COVID-19 Ag-RDT to clients and healthcare providers in Malawi, Nigeria, and Zimbabwe. Implemented occurred between April 2022 and June 2023, as COVID-19 transitioned from a global health emergency, with weekly cases across Africa declining from 6.5 million in January to 1.5 million by June 2023 [30]. The three countries represented unique settings with varying healthcare infrastructures, cultural contexts, and economic conditions [17–19]. Each country tailored its response to local contexts, focusing on community health workers and mobile solutions where needed.

#### Malawi.

Three use cases for COVID-19 testing were explored in Blantyre (rural and urban), Malawi: healthcare providers, Out-Client Department (OPD) and secondary distribution (December 2022-May 2023). The OPD use case provided provider-delivered testing to symptomatic individuals presenting to the facilities and unsupervised self-testing to confirmed cases for retesting and household contacts. Healthcare providers received options for provider-delivered testing or unsupervised self-testing according to facilities’ standard of care.

#### Nigeria.

Five use cases comprised: primary care facilities, community health facilities, workplaces, outbreak investigations, and secondary distribution (October 2022-May 2023). Provider-delivered Ag-RDT served diagnostic testing for symptomatic clients at primary care facilities, outreach activities at tertiary education centres and motor parks (i.e., local transport hubs), and testing within pharmacies and Patent Medicine Stores (PMS). Self-testing options were available at pharmacies and PMS, where clients self-selected for professional use or self-testing, with secondary distribution of self-test kits to household contacts via index clients.

#### Zimbabwe.

Four use cases included: sex workers, healthcare provider testing (both within the Key Populations (KP) programme implemented by CeSHHAR Zimbabwe), workplace use case, and clinic-based testing at New Start clinics (Population Solutions for Health). Implementation occurred in two phases: provider-delivered Ag-RDT (April 2022-January 2023) followed by combined provider-delivered and self-testing options (February 2023-June 2023). Phase one, the standard of care, provided testing to symptomatic individuals and confirmed cases contacts. Phase Two, offered testing choice to symptomatic individuals or contacts. KP Programme healthcare workers underwent weekly voluntary testing regardless of symptoms or exposure status.

Study participants were recruited from four facilities in Malawi, twelve in Zimbabwe, and twelve in Nigeria, encompassing both rural and urban geographical areas.

### Population

Participants comprised healthcare providers and healthcare seekers, including sex workers (Zimbabwe) and community members. The healthcare providers included peer educators, COVID-19 testers, clinicians, nurses and outreach providers. Sample size of 10–30 participants per use case were targeted in each country, based on the established literature indicating data saturation (when no more new themes or ideas emerge) typically occurs within this range [28].

Maximum variation sampling was employed to capture diverse perspectives on Ag-RDT implementation. Population selection utilised accessibility through established programmes, and representation of across different healthcare engagement points to enhance the transferability of findings. The 3ACP programme data and facility-based healthcare provider consultations informed identification of eligible participants within specified categories.

Inlcusion criteria encompassed adults aged 18 years and above, sex workers aged 16 years and older; healthcare providers, community pharmacists, PMS operators and their clients who were eligible to participate in the study, OPD attendees; and those willing and able to give written informed consent.

Recruitment procedures varied by setting. In static facilities, healthcare providers advertised the study prior to data collection, with interested individuals self-representing to the research team for information provision. For sex workers in Zimbabwe, the healthcare providers initiated contact and scheduled interview appointments. Experienced male and female social science researchers conducted interviews across all three country.

### Data collection and procedures

In-depth interviews were conducted at varying timepoints: immediately post-testing (Malawi and Nigeria) and four weeks post-testing (Zimbabwe). Pre-interview procedures included informed consent covering an overview of the purpose and process of the interview, data confidentiality and anonymity, and the need to audio record the interviews. Written informed consent was obtained and recorded. Interviews occured in the participants’ preferred language in a private and quiet place using a pre-developed semi-structured interview guide exploring key themes: acceptability perceptions, identified benefits and barriers, and user experience. Interviews averaged sixty minutes duration. Participants reimbursement was provided for their time contribution. Recruitment stopped uponing reaching data saturation in each use case.

### Data analysis

Interview audios were transcribed verbatim and translated by professional services, with research team verification against audio recordings for accuracy. Data analysis utilised NVivo software (version 14.23.4), employing inductive thematic approach [31] and constant comparison method [32] across countries, participant groups, and delivery models aligned with Louart’s determinants of acceptability.

Analytical procedures comprised iterative transcript review for data familiarisation and inductive code development. Country teams collaborated on initial transcript coding (n = 10), followed by consolidation, review, and refinement through merging and deletion (WSL and MP). The resulting comprehensive coding framework guided remaining transcript analyses by country-specific teams.

Disagreements were resolved through regular within-country team meetings facilitated by country leads, and weekly cross-country analytical meetings for pattern consolidation and thematic development. Constant comparison methodology enabled identification of patterns, differences, and category development across new and previously coded data. This iterative process allowed the teams to explore and contrast emerging themes within and across contexts, use cases and testing modalities. Analysis decisions were documented ensuring analytical rigour. Themes were refined against Louart’s framework through cross-country meetings, incorporating comprehensive team feedback for contextual analysis integration.

## Results

Qualitative data from 228 participants across Malawi, Nigeria, and Zimbabwe (Table 1) were analysed using Louart’s framework.

**Table 1:** Attributes of interviews (N = 228).

Country	Use case	Testing model	Participants	No of Interviews
**Malawi**	Healthcare workers	Self-testing	HSA, Lab techs, clinicians	16
		Provider testing	HSA, Lab techs, clinicians	10
	OPD	Provider testing	Community members	21
	Testers		HSA, Lab technicians, clinicians	12
	Index testing	Self-testing	Community testing	15
**Total**				**74**
**Nigeria**	Primary Health Care	Provider testing	Testers: Community health workers, Lab Technicians, Nurses	7
			Community members	8
	Community facilities (Patent Medicine Stores and Community pharmacies)	Dual offer of Provider testing and Self-testing	Testers: Community pharmacists and Patent Medicine Vendors	8
		Provider testing	Community members	3
		Self-testing	Community members	11
	Workplace	Self-testing	Office frontline workers	12
	Secondary distribution	Self-testing	Community members	3
**Total**				**52**
**Zimbabwe**	Sex workers testing	Phase 1	Sex workers	21
		Phase 2		8
	Health Worker	Phase 1	Nurses, Outreach workers, peer educators, micro planners	20
		Phase 2		5
	New Start Centre testing	Phase 1	General Population attendees	17
			Health workers: Nurses, Lab scientists	19
		Phase 2	General Population attendees	3
			Health workers: Nurses, Lab scientists	3
	Workplace testing	Phase 1	Workplace representatives, employees	6
**Total**				102

Cross-country analysis revealed patterns and variations in Ag-RDT acceptability, detailed below by domain (See Table 2 for the summary).

**Table 2:** Comparative matrix: summary of Ag-RDT acceptability across countries.

Louart Domain	Malawi	Nigeria	Zimbabwe	Cross-cutting Insight
**Compatibility**	Strong privacy/autonomy preference; integration with SRH	Vaccine complacency; private sector burden	Integrated testing increased access	Integration and cultural alignment influenced uptake
**Complexity**	Literacy barriers for self-testing	High provider confidence; some male client anxiety	Routine users adapted well (e.g., sex workers)	Literacy, gender, and health engagement history shaped the ease of use
**Perceived Advantages**	Improved rural access; task shifting reduced the burden	Decentralised testing; workplace convenience	Increased autonomy; minimised service disruption	Self-testing reduced the burden on systems and expanded reach
**Perceived Disadvantages**	Few, mostly logistical	Time burden in pharmacies; PCR refusal	Fewer reported limitations	Follow-up gaps and workload strain shaped provider experience
**Personal Emotion**	Pain reduction praised; some visual anxiety	Anxiety with swab; autonomy appreciated	Self-testing increased comfort	Emotional safety is key to adoption
**Social Influence**	Misinformation via social media, politicians, peer promotion	Political distrust, peer influence mixed	Peer trust encouraged uptake	Trust, political narratives, and social media shaped perceptions and behaviours.

### Compatibility

Ag-RDT demonstrated strong compatibility across all three contexts, reshaping COVID-19 testing access by addressing logistical and socio-cultural barriers that had constrained equitable testing. However, compatibility manifested differently across countries and populations.

The expedited turnaround time represented a universal compatibility strength across all contexts, though its impact varied by healthcare infrastructure. In Malawi’s resource-constrained rural setting, the 30-minute result window eliminated the burden of return visits:

*“The test was done quickly such that I waited for 30 minutes to get my test results as opposed to the test* [PCR test] *where people were told to come and get their test results the following day.”* – **Malawi_001_OPD client**

Self-testing options resonated universally with privacy preferences but demonstrated heightened significance amongst marginalised populations experiencing COVID-19 stigma. In Zimbabwe and Malawi, where COVID-19 stigma was particularly pronounced, self-testing provided a sense of control and autonomy. Self-testers performed the tests and interpreted the results privately, as reported by a participant in Zimbabwe who self-tested:

“*I was really comfortable because it does not involve anyone near you*.” – **Zimbabwe_003_Community member**

Cross-country analysis revealed critical differences in integration success. Public-sector clinics across all countries reported success integrating Ag-RDT into routine services. In Zimbabwe, Ag-RDT was integrated with Sexual Reproductive Health (SRH) services, eliminating multiple facility visits and reducing exposure risks. This was often achieved with minimal additional training or resources, fostering institutional support and promoting provider and client acceptance. A self-tester from Zimbabwe explained:

*“…they were able to get the services at one go rather than …previously they would go to Edith Opperman (a public sector health facility) to be tested for COVID then come back to get services from here while they are negative.”* – **Zimbabwe_02_Provider_sex workers facility**

Whilst Nigeria’s private-sector Patent Medicine Store model, though expanding access, created workflow tensions not observed in public-sector implementations. According to one provider:

*“As a patent store owner and an auxiliary nurse, I put other customers and clients on hold until the testing period ends. Some days I have a lot of clients who want to buy drugs... I must keep them waiting because I must conduct a provider-delivered COVID-19 testing... it takes about 20-30 minutes to conduct the test.”* – **Nigeria_006_Provider_Auxiliary nurse**

The waning perceived relevance of COVID-19 testing after the official pandemic declaration emerged as a universal challenge, subsequently impacting technological acceptability. This suggests that while participants found the technology acceptable, its timing may have been less optimal. As a Nigerian provider noted:

*“I have a few of the clients declining to do the Ag-RDT COVID-19 test. One of the main reasons I have found is that they said the COVID-19 outbreak has ended so there is no need to test”* **Nigeria_007_Provider_provider-assisted and self-testing**

### Perceived complexity

Ag-RDT, including self-testing, were user-friendly and accessible, though literacy mediated complexity perceptions. Clear instructions, provider demonstrations, and simple sample collection methods enabled confident use amongst providers and clients as stated below:

*“I have been well-trained and have the required skills to conduct the COVID-19 Ag-RDT test. Also, I am motivated and passionate about testing my community members to ensure there is no COVID-19 transmission in my community.”* **– Nigeria_004_Provider_provider-assisted and self-testing**

However, educational disparities created accessibility gaps, particularly in Malawi and Zimbabwe, where participants noted that written instruction could have challenged those with limited formal education:

*“Conducting the self-test with the instruction leaflet was easy, although it is for educated people. It might be difficult for people who are not educated, so I would suggest the language on the leaflet is simplified.”* **– Malawi_005_Index client**

This suggests the instructions were not adapted to ensure equitable access across diverse populations.

### Perceived advantages

Self-testing option emerged as a powerful approach to promote equity through the intervention, addressing geographical and mobility barriers that had constrained PCR testing access. Nigerian and Malawian participants particularly highlighted accessibility improvements for the elderly and disabled populations:

*“Some elderly people wanted to test for COVID-19 but did not because they could not go to the hospital due to the distance constraint.” Many people wanted to test but need access to PCR testing centres in the FCT [Federal Capital Territory], as we have very few PCR COVID-19 testing centres. However, with the introduction of self-testing, especially in pharmacy, many people have been testing to know their COVID-19 status.”* – **Nigeria_014_Provider**

*“It* [self-testing] *will help people with disabilities and the elderly...”* – **Malawi_003_Index Client**

Task-shifting to community health workers and self-testing reduced facility burdens, with benefits most pronounced in Malawi. The benefits included vehicle fuel savings, workload reductions, and reduced foot traffic for routine and contact testing. A provider noted:

*“It* [self-testing] *has reduced and eased the burden because we are saving fuel, and our workload is reduced.”* – **Malawi_003_Provider**

In occupational settings in Nigeria and Zimbabwe, workplace self-testing prevented productivity losses while enabling routine surveillance, safety and informing timely response actions. Providers in Nigeria and Zimbabwe commented that

*“We are now self-testing regularly; we conduct the test every week unlike testing for COVID-19 at the clinic.”* – **Nigeria_001_Provider**

*“As for me, self-testing was not disruptive but the journey to Chitungwiza general hospital was disruptive because it took my work time*” **Zimbabwe_003_Workplace self-tester**

### Perceived disadvantages

Exclusive to Nigeria, high confidence in self-test results created adherence challenges for confirmatory testing protocols, where PCR confirmation was required for positive cases. According to one provider:

*“The clients are very confident about the outcome of their self-test results. The positive cases we had in this pharmacy have always complied with our post-test counselling of self-isolation. Even when we asked them to go for a PCR test confirmation, they refused most of the time and told us that the self-test result outcome was enough for them.”* – **Nigeria_009_Provider_Pharmacist**

The confirmatory test was not in the Malawi and Zimbabwe study protocols.

### Personal emotions

Ag-RDT initiated positive emotional responses, alleviating the discomfort previously associated with PCR testing across all countries. A predominant response to PCR testing was anxiety and fear, often due to perceptions of a painful procedure, which deterred many from seeking testing.

*“COVID-19 testing is very painful because, during testing, a swab is inserted deep into the nostrils.”* **Zimbabwe_01_Sex worker**

The less invasive nature of Ag-RDT consistently enhanced acceptability. As one Malawian participant noted after experiencing both PCR and Ag-RDT methods:

*“It wasn’t my first time testing for COVID. I also got tested in 2021. They put something in my nose, and when it reached my throat, it was painful… However, today, it was a simpler and less painful experience.”* **– Malawi_005_OPD client**

This comparative assessment highlights how procedural modifications facilitated a shift in emotional response, generating comfort and reducing negative perceptions even amongst individuals with unpleasant testing experiences.

Self-testing options further increased autonomy in the testing process, contributing to overall comfort and emotional well-being. Nigeria and Zimbabwean participants elaborated on this preference, stating:

*“Aah, it’s much better doing it yourself in your comfort without fear, self-testing is good.”* – **Nigeria_01_Client_Self-testing**

*“…the method of self-testing is good, it is good because you will not hurt yourself*” **Zimbabwe_01_Community member self-testing**

Anticipatory anxiety persisted during provider-administered tests, often triggered by visual cues, suggesting that procedural modifications alone cannot eliminate all testing-related anxieties. One participant described the testing process saying:

*“I felt seriously frightened as she was inserting the swab into my nostril… Though it was not painful, just sighting someone trying to insert something into your nostril could make you feel very anxious and frightened…”* **– Nigeria_004_Client**

### Social influence

Social influence operated through contradictory mechanisms across all contexts, with social networks simultaneously facilitating and hindering acceptability. Clients expressed these influences, though notably, healthcare workers did not report experiencing similar social pressures.

Positive peer influence emerged through experiential testimonies with experienced individuals becoming community advocates. A participant in Malawi described how their explanation of self-testing advantages inspired another community member to take the test:

*“I told her that the type of testing that I did was different from the other type of testing that a lot of people are familiar with, and this motivated her to come and test for COVID-19.”* **Malawi_05_OPD client**

However, misinformation primarily through social media, infilled fears that tests were tools for viral transmission. Participants in Malawi and Zimbabwe shared how people refused to test, believing that it would infect them with the virus:

*“He refused because of what people believe that self-testing for COVID-19 is one of the approaches that are being used to spread COVID-19, so I just decided not to force him.” –* **Malawi_01_Index client**

*“Yeah, because usually I was skeptic about this whole thing, because you know outside there, they will tell you no, you don’t actually have COVID, but now they are actually inserting COVID”* – **Zimbabwe_03_Community member**

The idea that self-testing kits could be weaponised against communities reflects a lack of understanding of the technology and the broader societal anxieties about external interference in community health as described by a healthcare worker:

*“Yeah, these are the messages that people are sharing in the villages, and it’s happening that some people are showing symptoms of Corona, but they don’t want to come to the hospital because they are afraid that if they come to the hospital, they will be killed, so they stay back in the village.”* – **Malawi_007_Health worker**

Political narratives fundamentally challenged testing legitimacy, with community members viewing COVID-19 as governmental manipulation. This political framing was evident in Malawi and Nigeria:

*“Many even reject it in your face that they do not have COVID-19, and others believe that COVID-19 does not exist, that it was a prank for government officials to make money.” –* **Nigeria_006_Provider_provider-assisted and self-testing**

*“At the time, people were arguing against political parties. It is understood that politicians created this issue; the disease does not exist, but politicians are just speculating with the aim that some people’s political rallies should not be held.”* **Malawi_002_Tester**

Across all contexts, temporal shifts in risk perception reduced testing urgency as COVID-19 became normalised as an ordinary illness, creating additional barriers to Ag-RDT uptake. Providers in Malawi and Zimbabwe noted:

*“Yes, now they (community members) regard it (COVID-19) as the regular flu.” –* **Malawi_09_Index client**

*“...I think people now are sort of getting complacent thinking COVID is gone.”* – **Zimbabwe_09_Health worker**

## Discussion

This qualitative study explored COVID-19 Ag-RDT acceptability across Malawi, Nigeria, and Zimbabwe. Ag-RDT achieved high acceptability amongst clients and healthcare providers, driven by technological advantages that addressed key PCR testing barriers. However, acceptability was constrained by implementation timing, social-political dynamics, and healthcare system capacity differences. While these results confirm acceptability patterns from other rapid diagnostic implementations, they reveal how acceptable technologies face implementation challenges that limit population-level relevance.

Our findings echo acceptability patterns documented in other RDT implementations, particularly HIV self-testing programmes across Africa [9–12,33,34], However, our study provides valuable LMIC-specific evidence for COVID-19 Ag-RDT, demonstrating how these universal acceptability factors manifest in diverse African healthcare contexts during a pandemic response. Systematic review identified perceived accuracy, ease of use, confidentiality, privacy, and convenience as universal drivers [10], and a mixed methods study in Malawi found self-testing particularly attractive to underserved populations [11]. Self-testing here successfully reached underserved groups while reducing facility burdens. Public-sector integration succeeded across countries, though Nigeria’s private sector faced operational constraints, extending findings on resource challenges facing private providers [35]. This consistency suggests certain acceptability factors may be relatively stable across rapid diagnostic applications in similar settings [35–40].

Despite high Ag-RDT acceptability, contextual factors constrained relevance and uptake. Implementation timing emerged as a critical constraint. Our study occurred during declining COVID-19 transmission when many questioned testing necessity. This temporal dimension distinguishes our findings from Bresser et al.‘s COVID-19 self-testing evaluation in Lesotho and Zambia [16] and Mukoka et al.’s Malawi study [15], both conducted during active transmission when testing urgency remained high. Rapid diagnostic deployment emphasises speed [4,5] but assumes acceptable technologies will be utilised when available. Our findings suggest technologies deployed after perceived urgency declines may face reduced uptake regardless of acceptability characteristics, a phenomenon undertheorised in pandemic preparedness literature. This timing effect could have affected acceptability through reduced perceived risk, pandemic fatigue, and competing health priorities [41].

Social and political factors significantly influenced acceptability. Historical medical mistrust, compounded by COVID-19-specific misinformation spread through social media platforms, created barriers transcending individual technology preferences [42–46]. This mistrust compounded by limited public understanding of the technology itself and a broader absence of credible health information, even at end of the pandemic [44–46]. While social media’s role in fueling Ebola-related misinformation is well-documented [47,48], our findings expand this understanding by demonstrating how online narratives were filtered through and amplified by existing local mistrust in diagnostic technologies. This dynamic underlines the need for culturally resonant, timely health communication strategies where community leaders and local influencers play pivotal roles in either legitimising or delegitimising testing technologies [49,50]. Implementation strategies must explicitly account for social media’s role in shaping technology acceptance and proactively engage religious and political leaders as key stakeholders [47,48] in these contexts.

COVID-19 Ag-RDT demonstrate sensitivity of 34–88% and specificity >95% compared to RT-PCR, with performance dependent on viral load and symptom presence [51,52]. This lower sensitivity limits their use for surveillance and positions them as complementary rather than replacement diagnostic tools [51]. Despite these limitations, participants expressed high confidence in results, with some Nigerian participants declining confirmatory PCR testing after positive results. The varying approaches to confirmatory testing following a positive self-test result warrant careful consideration in evaluating the intervention’s implementation. While the study protocols included confirmatory testing requirements primarily for research purposes, this additional step was not consistently aligned with real-world practices or WHO COVID-19 testing guidelines [7,8]. Hence, rather than viewing particpants’ refusal of confirmatory tests as reluctance or non-compliance, it represents a reasonable response given the testing landscape at the time.

These findings must be understood within the broader global health inequities context. While high-income countries rapidly implemented COVID-19 self-testing programmes [53], African nations faced significant delays. This mirrors earlier HIV epidemic experiences and raises critical questions about preparedness, equity, and the timeliness of global health responses [54]. Addressing these inequities will require faster access to new technologies and more inclusive and context-sensitive deployment mechanisms in LMIC.

### Relevance of Louart’s acceptability framework

Louart’s acceptability framework [25] provided useful organizing categories through six domains, enabling systematic examination of technological characteristics, organisational contexts, individual behaviours. The framework’s helped identify facilitators to technology adoption, understand the contextual implementation constraints revealing that successful intervention extends beyond technological efficacy.

While Louart’s domains organised findings effectively, the framework does not adequately account for temporal dynamics, macro-political forces, or resource constraints. The social influence domain captures peer effects but not political narratives or social media amplification that fundamentally shaped acceptability in our contexts. The compatibility domain examines fit with existing practices but not resource competition or operational differences between healthcare sectors. Implementation timing has no explicit place in the framework. Future research might benefit from frameworks explicitly integrating epidemic phase, resource constraints, and socio-political context as core determinants rather than background factors [26].

### Strengths and limitations of the study

The study’s strengths include an in-depth exploration of client and healthcare provider perspectives and a systematic analysis of acceptability across different contexts using theoretical framework revealing how diagnostic technologies can be effectively integrated into diverse healthcare environments and user acceptance.

The study had a few limitations. Selection and social desirability biases may have influenced responses as participants who agreed to participate may have differed systematically from those who declined, potentially overrepresenting individuals with more favourable attitudes toward testing technologies. These was mitigated through confidentiality assurance, trained local interviewers, and triangulating across diverse participant groups and settings. Implementation timing during declining transmission likely created favourable conditions. Acceptability measured during low urgency may not predict uptake during active outbreaks when different dynamics including, higher anxiety, greater system strain, and urgency-driven decision-making, would operate. The qualitative design, while appropriate for understanding experiences, cannot establish causation or predict population-level outcomes. Given these limitations, conclusions regarding high acceptability should be interpreted cautiously, particularly for active outbreak scenarios.

## Conclusion

COVID-19 Ag-RDT achieved high acceptability amongst clients and healthcare providers across Malawi, Nigeria, and Zimbabwe, although acceptability was constrained by implementation timing, social-political dynamics, and healthcare system capacity differences.

This study confirms acceptability patterns observed in other RDT implementations while providing essential context-specific evidence for COVID-19 Ag-RDT in three African countries. Our findings emphasise positioning Ag-RDT as complementary to RT-PCR and NAAT tests, in resource-constrained settings. Successful deployment strategies require early implementation, community engagement, healthcare system capacity aligned with operational demands. Implementation strategies must explicit attention to how social dynamics and resource constraints shape technology adoption beyond individual user preferences.
